# Structural Determinants and Repair of Membrane Microdomains in Dendritic Cell-Mediated Antitumor Immunity: An Integrative Mechanistic Synthesis

**DOI:** 10.3390/ijms27052305

**Published:** 2026-02-28

**Authors:** Ramón Gutiérrez-Sandoval, Francisco Gutiérrez-Castro, Natalia Muñoz-Godoy, Ider Rivadeneira, Andy Lagos, Jordan Iturra, Francisco Krakowiak, Cristián Peña-Vargas, Matías Vidal, Andrés Toledo

**Affiliations:** 1 Department of Oncopathology, OGRD Alliance, Lewes, DE 19958, USAops@ogrdalliance.org (A.T.); 2 Department of Cancer Research, Flowinmunocell-Bioexocell Group, 08028 Barcelona, Spain; 3 Department of Outreach and Engagement Programs for OGRD Consortium, Charlestown KN0802, Saint Kitts and Nevis; 4 Bioclas, Concepción 4030000, Chile

**Keywords:** dendritic cells, antitumor immunity, membrane microdomains, lipid rafts, immune synapse, cancer immunotherapy, tumor microenvironment, phospholipoproteic complexes (PLPC), raft repair, dendritic cell-derived exosomes

## Abstract

Durable responses to cancer immunotherapy remain restricted to a subset of patients, highlighting persistent gaps in understanding immune failure mechanisms. Dendritic cells (DCs) serve as the critical bridge between antigen recognition and adaptive immune activation, yet conventional molecular models centered on discrete components fail to fully explain heterogeneous therapeutic outcomes. This integrative mechanistic synthesis proposes that DC-mediated antitumor immunity is governed by higher-order structural determinants, including membrane microdomain organization, spatial compartmentalization of signaling, and temporal integration of antigenic and co-stimulatory cues. These features determine whether antigen presentation leads to effective T-cell priming or dysfunctional states such as exhaustion or anergy within the tumor microenvironment. By reanalyzing our validated 2025 experimental pipeline alongside high-impact contextual literature, we identify emergent properties of immune competence that transcend linear molecular interactions. The resulting framework distinguishes structurally mediated failure modes from classical resistance paradigms, providing a coherent non-reductionist explanation for variability in immunotherapy efficacy. Membrane raft repair is positioned as a key promising structural condition for effective immune integration, with direct relevance to translational and regulatory contexts involving non-pharmacodynamic platforms and New Approach Methodologies (NAM)-aligned evaluation strategies. This work proposes an integrative mechanistic framework to guide future hypothesis-driven studies and clinical advancement of DC-based approaches.

## 1. Introduction

Despite transformative progress in cancer immunotherapy, durable antitumor responses continue to be achieved in only a minority of patients [1]. Molecularly targeted therapies and immune checkpoint inhibitors have revolutionized clinical practice [2], yet their variable efficacy underscores ongoing limitations in comprehending immune failure within the cancer context [3]. While target identification and pathway modulation remain central foci, emerging evidence increasingly points to higher-order organizational constraints within immune cells as pivotal yet underappreciated determinants.

Dendritic cells (DCs) occupy a central role in antitumor immunity, linking antigen recognition to adaptive immune activation [4]. Classical models have predominantly focused on discrete molecular elements—receptors, ligands, cytokines, and transcriptional programs—as independent drivers of immune responses. However, accumulating observations indicate that effective immune competence depends critically on the spatial organization of membrane components, the architectural integrity of intracellular signaling platforms, and the precise temporal coordination of antigenic and co-stimulatory signals [5]. These structural parameters collectively determine whether antigen presentation results in robust T-cell priming or functional impairment [6].

In the tumor setting, this structural dependency becomes particularly evident. Tumor-associated immune dysfunction frequently arises in the absence of overt molecular defects, genetic alterations, or dominant inhibitory ligands. Clinical and experimental data commonly document states of exhaustion, anergy, or non-responsiveness that conventional resistance mechanisms do not adequately explain [7]. These findings suggest the existence of structural failure modes, in which essential molecular machinery persists but lacks the organizational coherence required for integrated signal processing and effective immune orchestration [8].

The plasma membrane of immune cells forms a dynamic mosaic organized into nanoscale lipid rafts—cholesterol- and sphingolipid-enriched ordered (Lo) domains separated from disordered (Ld) regions through phase separation. Rafts function as platforms for protein clustering and signal amplification. In DCs, they stabilize major histocompatibility complex class II (MHC-II) and cluster of differentiation 86 (CD86) for efficient antigen presentation; disruptions induced by tumor microenvironment (TME) stressors, such as oxidative stress, lipid peroxidation, cholesterol depletion, and transmembrane potential alterations, lead to fragmented domains, unstable immune synapses, abortive signaling, and tolerance induction [9].

Membrane microdomain repair therefore represents a powerful non-reductionist intervention. Vesicle-derived platforms, including dendritic cell-derived exosomes (DEXs) and ultrapure phospholipoproteic complexes (PLPCs), deliver phospholipids and anchored proteins that reintegrate into compromised membranes, restoring ordered phase stability, signaling cluster integrity, synapse geometry, and electrochemical homeostasis. This repair process operates as an emergent property of structural reintegration rather than direct molecular agonism [10].

This concept is supported and extended by our sequential experimental pipeline published in 2025, which provides direct empirical evidence for the functional impact of membrane microdomain repair in DC-mediated immunity [11,12,13]. Specifically, these studies demonstrate that ultrapure PLPC and DEX platforms restore raft integrity and immune synapse stability ex vivo [14,15,16,17], enable real-time phenotypic stratification of tumor responses without cytotoxicity [18,19], offer traceability of biological activity without clinical exposure [11], preserve immunological fingerprints for scalable precision therapy [15], and establish multistage monitoring protocols for quality control and immune profiling [20]. By integrating these validated results with contextual high-impact literature, the present synthesis formalizes membrane raft repair as a unifying structural determinant that explains variability in immunotherapy outcomes beyond traditional molecular paradigms.

The objective of this article is to provide an integrative mechanistic synthesis of DC function grounded in structural biology and immune system organization. By systematically integrating our previously published experimental evidence from the 2025 pipeline with contextual high-impact literature, we formalize a coherent structural framework that unifies DC-mediated immune competence, positions membrane microdomain repair as a central determinant, and offers a non-reductionist explanatory model for current limitations in cancer immunotherapy.

## 2. Scope, Evidence Base and Methodological Framework

This article presents an integrative mechanistic synthesis that reinterprets and structurally reanalyzes previously validated scientific evidence to establish a unified conceptual framework for dendritic cell-mediated antitumor immunity [11]. The synthesis builds upon a deliberate sequential experimental pipeline conducted by our research group, as documented in peer-reviewed publications from 2025.

The primary evidence base consists of five interconnected studies published in 2025 that form a deliberate sequential experimental pipeline conducted by our research group. These studies collectively deliver cross-validated functional readouts of raft integrity, immune synapse stability, cytokine polarization profiles, batch-to-batch reproducibility, and regulatory-compatible documentation metrics, providing the empirical foundation for the present structural synthesis. Specifically: (1) proteomic and structural characterization of an ultrapurified phospholipoproteic complex (PLPC) as a scalable immunomodulatory platform for reprogramming immune suppression in metastatic cancer [18]; (2) evaluation of phospholipid-rich DC vesicles with preserved immune fingerprints as a stable and scalable platform for precision immunotherapy [15]; (3) real-time functional stratification of tumor cell lines using a non-cytotoxic phospholipoproteomic platform in a label-free ex vivo model [19]; (4) development of an ex vivo traceability platform for phospholipoproteomic formulations providing functional evidence without clinical exposure [11]; and (5) design of a multistage monitoring protocol for dendritic cell-derived exosome (DEX) immunotherapy as a conceptual framework for molecular quality control and immune profiling [20].

Contextual high-impact literature has been selectively incorporated to situate our findings within the broader field, contrast molecular and structural paradigms, and support the generalizability of the proposed framework [6,7,8,9,10,21,22,23,24,25].

The methodological approach is interpretative and non-experimental. Previously published results are systematically reexamined through a structural lens that emphasizes spatial microdomain organization, membrane architecture, intracellular trafficking pathways, and temporal signal coordination as primary determinants of immune competence. Recurring organizational patterns are abstracted across independent experimental contexts to achieve mechanistic coherence and conceptual unity.

The synthesis prioritizes explanatory depth and integrative insight over exhaustive literature coverage, delivering a stable interpretative framework suitable for guiding experimental design, translational development, and regulatory evaluation of non-pharmacodynamic immunomodulatory platforms [20].

## 3. Structural Organization of Dendritic Cells and Immune Competence

Dendritic cells function as highly organized multicomponent systems in which immune competence emerges from the coherent integration of structural elements across multiple hierarchical scales [21]. Effective antitumor immunity requires precise spatial arrangement of membrane constituents, intact intracellular trafficking networks, and coordinated assembly of signaling platforms at the immunological synapse.

At the plasma membrane level, DCs exhibit dynamically regulated microdomains that spatially constrain receptor clustering, co-stimulatory molecule engagement, and downstream signal propagation. Disruption of this nanoscale organization decouples receptor ligation from productive intracellular signaling cascades, even when individual molecular components remain intact [22].

Antigen presentation itself is structurally dependent: efficient peptide loading onto MHC molecules, intracellular trafficking of MHC–peptide complexes, surface display, and formation of stable immunological synapses all rely on coordinated interactions between the plasma membrane and the underlying cytoskeleton. Impairment at any of these interfaces can result in antigen recognition without effective T-cell priming—a hallmark of tumor-associated immune dysfunction [23].

Intracellular organization governs antigen processing, vesicular routing, and delivery of contextual signals that modulate the quality of the immune response. Temporal coordination further regulates signal persistence, receptor recycling, and feedback mechanisms, ensuring synchronized activation and appropriate polarization [24].

**Figure 1:**
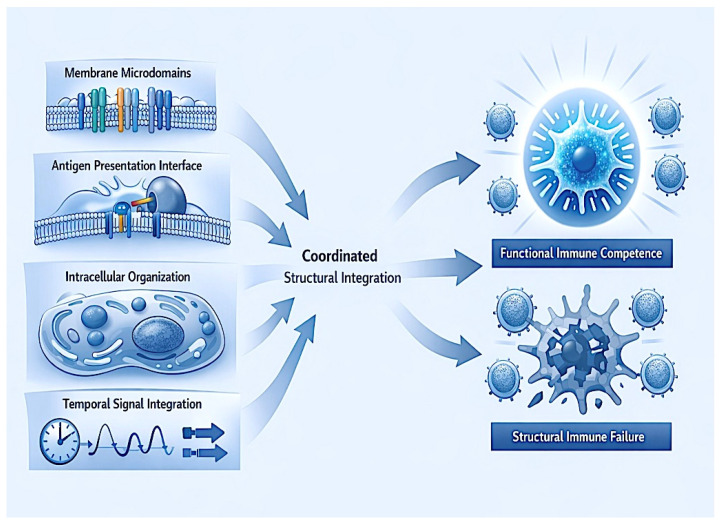
Structural organization of dendritic cell-mediated immune competence. Schematic illustration depicting the coordinated interplay of membrane microdomains, antigen presentation interfaces, intracellular vesicular routing, and temporal signal synchronization, leading to either functional competence or structural failure.

In the tumor microenvironment, chronic stressors progressively compromise these structural layers without necessarily eliminating core molecular machinery, leading to a loss of organizational coherence and consequent functional failure [25]. This structural perspective provides a mechanistic basis for understanding why DCs exposed to similar antigenic and inflammatory stimuli can produce divergent immunological outcomes depending on the integrity of their membrane and intracellular architecture [26] (Figure 1; Table 1)

## 4. Microdomain-Level Signal Integration in Antigen Presentation

Antigen presentation by dendritic cells relies on the precise integration of antigenic, co-stimulatory, and contextual signals within spatially confined membrane microdomains. These specialized domains serve as dynamic organizing platforms that concentrate transmembrane receptors, adaptor proteins, and signaling enzymes, thereby determining the amplitude, duration, and qualitative nature of the resulting immune response [27].

In DCs, the spatial arrangement of MHC–peptide complexes, costimulatory molecules (such as cluster of differentiation 80 (CD80) and cluster of differentiation 86 (CD86)), and pattern recognition receptors within microdomains enables cooperative signaling that cannot be replicated by dispersed molecular interactions alone. This spatial constraint generates non-linear signal amplification through threshold-dependent effects, positive feedback loops, and allosteric interactions among clustered components [28].

Signal integration at the microdomain level is inherently non-linear and temporally gated. The persistence of receptor clustering, the duration of co-stimulatory engagement, and the precise sequencing of downstream pathway activation collectively shape whether antigen encounter leads to productive activation or incomplete/tolerogenic responses [29]. Microdomains regulate these parameters by restricting lateral mobility of receptors, stabilizing transient signaling complexes, and facilitating sequential recruitment of intracellular mediators [30].

Disruption of microdomain architecture—whether through lipid peroxidation, cholesterol depletion, or alterations in transmembrane potential—results in asynchronous or fragmented signaling, even in the presence of functionally intact molecular machinery [31]. This structural decoupling provides a mechanistic explanation for divergent functional outputs observed in DCs exposed to comparable antigenic and inflammatory stimuli, depending on the preservation of membrane order [32].

Within tumor microenvironments, chronic biophysical and biochemical stressors progressively compromise microdomain organization, reconfiguring spatial relationships among signaling constituents without eliminating them. The consequence is antigen presentation that becomes structurally uncoupled from effective T-cell priming, favoring incomplete activation, transient responses, or induction of tolerance [33].

Thus, antigen presentation constitutes a fundamentally microdomain-governed process in which immune outcomes are determined by the degree of structural integration rather than molecular abundance alone [34]. This perspective unifies seemingly disparate clinical phenotypes and establishes microdomain integrity as a foundational determinant of DC function [35].

## 5. Structural Failure Modes in Dendritic Cell-Mediated Immunity

Immune dysfunction in cancer frequently persists despite preservation of essential molecular machinery, indicating the operation of structural failure modes at the cellular level. These failure modes arise from progressive loss of organizational coherence, resulting in impaired spatial–temporal signal integration and ineffective immune orchestration [36].

A primary failure mode involves microdomain disorganization. When spatial clustering of antigen-presenting and co-stimulatory molecules is disrupted, receptor ligation fails to translate into coherent downstream signaling cascades. This decoupling manifests clinically as partial T-cell activation, insufficient clonal expansion, defective effector differentiation, or premature exhaustion, even when canonical pathways remain functionally intact [37].

A second major failure mode concerns temporal misalignment of immune signals. Productive DC activation requires tightly orchestrated sequencing of antigen recognition, co-stimulation, and cytokine-driven polarization. Structural perturbations that alter receptor mobility, signal persistence, or vesicular trafficking disrupt this temporal precision, yielding responses that are abortive, disproportionate, or short-lived [38].

Intracellular architectural compromise represents an additional layer of dysfunction. Alterations in endosomal sorting, vesicular transport, and organelle positioning impair antigen processing and contextual presentation without abolishing molecular functionality. Such disorganization can result in antigen display devoid of appropriate co-stimulatory or polarizing context, actively promoting tolerance induction rather than immunity [39].

Tumor-associated chronic stressors—metabolic competition, persistent antigen load, and immunosuppressive cues—exert cumulative biophysical effects that erode structural coherence over time. The progressive and heterogeneous nature of this erosion explains why immune dysfunction often develops gradually and variably across patients, even with comparable tumor antigenic profiles or molecular biomarkers [40].

These structural failure modes offer a unifying explanation for clinical variability in immunotherapy responsiveness: patients with similar molecular characteristics exhibit markedly different immune responses and therapeutic outcomes due to differences in cellular organizational integrity [41]. This perspective shifts the diagnostic and therapeutic focus from isolated molecular defects to restoration of higher-order structural coherence [42].

**Figure 2:**
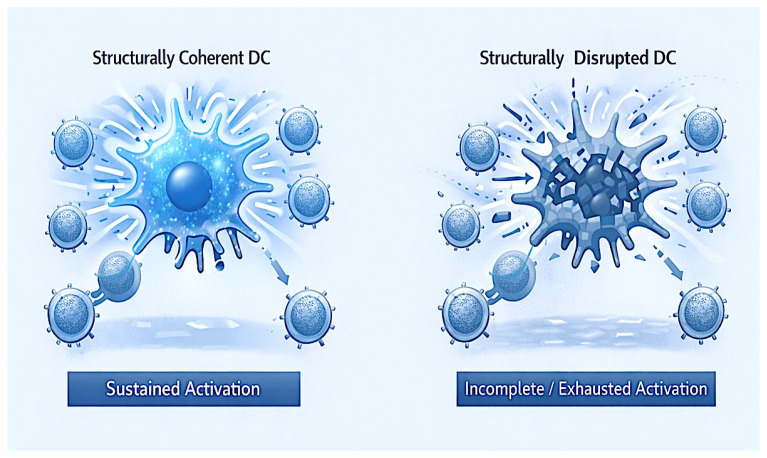
Structural failure modes underlying dendritic cell-mediated immune dysfunction. Schematic contrasting coherent organizational states enabling sustained activation with disrupted states characterized by spatial disorganization, temporal misalignment, and loss of functional coherence, leading to incomplete priming or exhaustion.

The strength of evidence for these structural failure modes derives primarily from controlled ex vivo and biophysical models in our 2025 pipeline [11,15,18,19,20], where raft disruption, synapse instability, and temporal misalignment were directly measured without confounding in vivo variables. Clinical correlation remains preliminary but supportive, as similar organizational defects have been observed in tumor-infiltrating DCs from “cold” tumors [21,27]. While genetic and metabolic mechanisms are more extensively validated in patients [43,44], structural determinants provide a mechanistic explanation for cases where molecular components are preserved yet function fails, warranting further translational studies to assess causality in human cohorts. (Table 2; Figure 2)

## 6. Integrative Structural Framework for DC-Driven Antitumor Immunity (Synthesis and Interpretation)

DC-mediated antitumor immunity emerges from the coordinated interplay of spatial organization, temporal signal integration, and cellular architectural state, as consistently demonstrated by the convergent evidence from our sequential experimental pipeline published in 2025 [11,15,18,19,20]. While prior literature has described individual aspects of membrane microdomains in DCs [9,10,33], the present synthesis integrates these elements into a unified non-reductionist framework centered on raft repair as an emergent hub.

Within this integrative framework, dendritic cells operate as non-linear integrative systems. Membrane microdomains provide the primary spatial scaffold for cooperative receptor signaling and threshold-dependent amplification [45]. Intracellular architecture ensures appropriate antigen routing, vesicular trafficking, and contextual signal delivery. Temporal dynamics govern the sequence, duration, and synchronization of cues, enabling precise feedback regulation and polarization. Immune competence arises as an emergent property of coherent integration across these dimensions, rather than from the isolated activity of individual molecular components [46].

Non-linearity is a defining characteristic of the system: minor perturbations in microdomain stability, cytoskeletal linkage, or trafficking kinetics can produce disproportionately amplified functional consequences, while preservation of molecular components in the absence of structural coherence fails to guarantee effective activation [44]. This property mechanistically accounts for the observed divergence in immunological outcomes under similar antigenic or inflammatory conditions.

Context dependence is equally intrinsic. Metabolic pressures, microenvironmental factors, and chronic antigenic load dynamically reshape cellular architecture, altering signal integration capacity without altering molecular identity. This intrinsic variability provides a coherent explanation for inter-patient heterogeneity in immunotherapy responsiveness, even among individuals with comparable tumor antigenic profiles or molecular biomarkers.

The framework fundamentally shifts the analytical focus from molecular sufficiency to structural integration. It clarifies why immune activation can fail despite intact signaling machinery and why effective therapeutic modulation requires restoration of organizational coherence beyond target-specific interventions [47].

From a translational perspective, structural determinants offer measurable, system-level criteria that enhance interpretability across preclinical, clinical, and regulatory contexts without presupposing specific mechanisms of action. This alignment supports evidence-proportionate assessment of emerging non-pharmacodynamic platforms, including vesicle-derived formulations such as PLPC and DEX, as documented in our recent series [48].These interpretative implications are summarized in Table 3.

**Figure 3:**
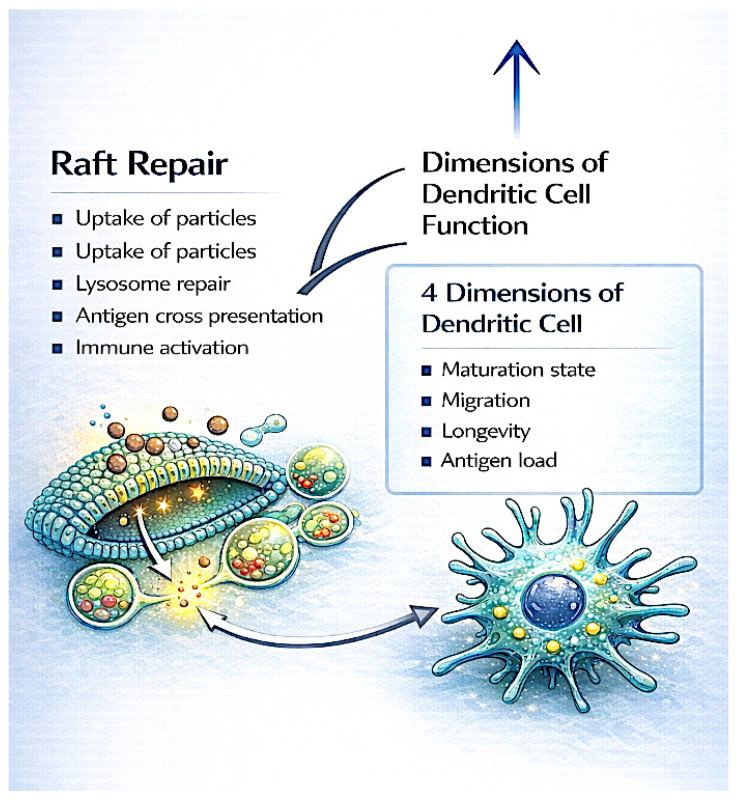
Raft repair as an integrative structural hub coordinating dendritic cell functional outputs. Depicts membrane raft repair as a central bidirectional hub linking antigen uptake, maturation, migratory competence, and T-cell activation.

**Figure 4:**
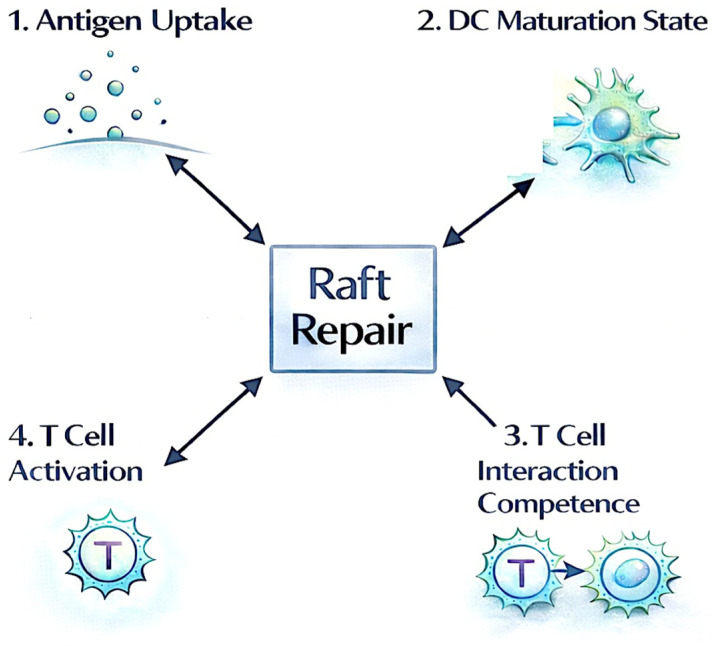
Raft repair as a non-linear structural hub integrating dendritic cell functional dimensions. Conceptualizes raft repair as the enabling condition for simultaneous, coordinated expression of key DC functions in a non-sequential, integrative manner.

The integrative structural framework presented herein conceptualizes raft repair as a central, non-linear hub that simultaneously coordinates multiple functional dimensions of dendritic cells, including antigen uptake, maturation state, T-cell interaction competence, and T-cell activation. Rather than following a sequential pathway, raft repair enables the emergent, integrative expression of these key processes, thereby restoring coherent immune competence and overcoming structural failure modes in the tumor microenvironment (Figure 3; Figure 4). This hub model underscores the pivotal role of membrane microdomain integrity as an enabling condition for effective DC-mediated antitumor immunity.

## 7. Regulatory and Translational Implications

Conventional regulatory evaluation of immunomodulatory agents prioritizes molecular target engagement and pathway-specific pharmacodynamics. While effective for classical drugs, these criteria provide limited resolution for platforms whose activity depends on higher-order structural restoration rather than direct receptor agonism or systemic exposure [49].

The structural framework presented here complements existing paradigms by offering reproducible, traceable, and mechanism-independent parameters—such as membrane organization, signal compartmentalization, and temporal coordination—that align with New Approach Methodologies (NAM) and non-pharmacodynamic evaluation strategies. This alignment supports proportionate, evidence-based assessment of vesicle-derived and biomimetic platforms without necessitating traditional clinical exposure endpoints [50].

Recent clinical and translational advances in DC-based immunotherapy, including dendritic cell-derived exosomes (DEX), underscore the growing interest in cell-free platforms to overcome limitations of whole-cell approaches. For instance, tumor antigen-loaded DEXs have shown safety and feasibility in early-phase trials for non-small cell lung cancer (NSCLC) and melanoma, with evidence of T-cell and NK-cell activation, albeit modest objective responses in some cohorts [13,14]. More recent reviews highlight the potential of DEX as a next-generation, scalable alternative, emphasizing their ability to preserve immunological fingerprints, induce cytotoxic T-lymphocyte responses, and target cancer stem cells while bypassing challenges associated with live DC vaccines [51,52]. Ongoing or recently discussed Phase II trials continue to explore DEX in maintenance settings post-chemotherapy in advanced NSCLC and other solid tumors, with promising signals for combination strategies that could synergize with structural interventions like raft repair to enhance immune synapse stability and overcome TME-induced dysfunction [51,52]. These developments align with our proposed framework by positioning DEXs not merely as antigen carriers but as structurally restorative platforms capable of membrane microdomain reintegration, warranting further hypothesis-driven integration in regulatory-aligned non-pharmacodynamic evaluations.

Transitionally, the framework reconciles frequent discrepancies between preclinical potency and clinical efficacy by attributing variability to differences in structural integrity rather than unexplained biological heterogeneity. Structural readouts thus enable more accurate contextualization, comparative analysis across platforms and indications, and improved patient stratification in real-world settings [53].

By emphasizing organizational coherence over isolated target modulation, the model facilitates regulatory flexibility for emerging non-pharmacodynamic interventions while maintaining scientific rigor and audit-ready documentation [54]. This is particularly relevant for scalable, cold-chain-independent formulations such as PLPC and DEX, which have demonstrated immunological consistency and phenotypic stratification capacity in our validated pipeline [11,15,18,19,20].

## 8. Limitations and Boundaries of the Present Analysis

This synthesis is deliberately constrained to our validated experimental corpus and selected high-impact contextual literature, with a clear emphasis on mechanistic coherence over exhaustive review. Consequently, it prioritizes structural determinants while assigning relatively less weight to alternative genetic, epigenetic, or purely tumor-intrinsic paradigms [55,56].

As a conceptual and interpretative work, it introduces no new primary data. Although supported by proteomic, biophysical, and functional evidence from controlled ex vivo models across our 2025 pipeline, the framework awaits large-scale clinical validation to establish causal generalizability in human subjects [57].

Far from supplanting molecular models, this structural perspective is intended to complement them by addressing emergent properties and higher-order failure modes that reductionist analyses may overlook. Future refinements could incorporate structured ex vivo traceability metrics, functional stratification algorithms (e.g., STIP), and multi-omics integration to further bridge mechanistic insight with translational reproducibility and regulatory alignment [58].

## 9. Conclusions and Conceptual Closure

This integrative mechanistic synthesis provides conceptual integration for our accumulated experimental evidence, establishing a structural paradigm for dendritic cell-mediated antitumor immunity. Effective immune activation emerges as the product of membrane microdomain organization, spatial signal compartmentalization, and temporal cue integration. Re-examination through this lens identifies structural failure modes—distinct from linear molecular defects—as central drivers of persistent limitations in immunotherapy, including T-cell exhaustion, anergy, and poor responses in immunologically “cold” or metastatic tumors [52].

The non-reductionist framework contrasts structural determinants with conventional resistance paradigms, providing a coherent mechanistic basis for heterogeneous outcomes in DC-based strategies. Leveraging advanced vesicular platforms such as ultrapure phospholipoproteic complexes (PLPC) and dendritic cell-derived exosomes (DEX), as documented in our validated 2025 pipeline [11,15,18,19,20], the model suggests biophysical membrane repair as a targeted approach to restore raft integrity, stabilize immune synapses, and reprogram immunosuppressive microenvironments without reliance on pharmacodynamic targets or systemic exposure [59].

These structural interventions align seamlessly with emerging regulatory pathways for non-pharmacodynamic platforms, where ex vivo traceability and functional stratification enable reproducible documentation of immune competence. Ultimately, durable antitumor immunity requires both molecular precision and architectural coherence within immune cells [60].

By formalizing membrane microdomain repair as a foundational enabling mechanism, this work furnishes a robust conceptual foundation to inform future translational development—including scalable, cold-chain-independent formulations and structured real-world monitoring protocols—thereby advancing DC-based immunotherapy toward greater predictability and efficacy against refractory malignancies.

## 10. Contextualization with Alternative Mechanisms and Final Perspectives

To provide a balanced and critical perspective, this structural framework is contextualized with alternative and competing mechanisms of DC dysfunction in cancer, including genetic alterations, metabolic reprogramming, transcriptional dysregulation, and epigenetic modifications [43,44,57,58]. Genetic mutations or copy number variations in DC-related genes (e.g., *IRF8*, *BATF3*) can impair differentiation or maturation [44], while metabolic competition in the tumor microenvironment (e.g., glucose or amino acid depletion) reprograms DC energy pathways, favoring tolerogenic phenotypes [43]. Transcriptional programs driven by STAT3, HIF1α, or Wnt/β-catenin signaling further suppress immunogenic functions [57], and epigenetic changes such as histone methylation or DNA hypermethylation silence co-stimulatory genes.

These mechanisms often operate in parallel or synergistically with structural disorganization. However, our synthesis highlights cases where core molecular machinery remains intact but fails due to loss of higher-order coherence (e.g., fragmented microdomains despite preserved MHC-II/CD86 expression) [11,15,18,19,20]. This structural layer offers complementary explanatory power, particularly in “cold” or refractory tumors where molecular defects are absent or insufficient to account for observed dysfunction [21,27]. By integrating these perspectives, the framework avoids reductionism and underscores the need for multi-level analyses in future studies (Table 4).

## Figures and Tables

**Table 1:** Structural determinants of dendritic cell-mediated immune competence: a summary of key membrane, intracellular, temporal, and system-level organizational features whose coordinated integration enables effective antitumor immunity. Disruption at any level can produce functional failure despite preservation of individual molecular components.

Structural Level	Key Organizational Features	Functional Consequence for Immunity	Failure Phenotype When Disrupted
Plasma membrane architecture	Microdomain organization, receptor clustering	Efficient antigen recognition and signal amplification	Fragmented signaling, incomplete T-cell priming
Antigen presentation interface	MHC stability, synapse integrity	Sustained T-cell activation	Abortive or tolerogenic responses
Intracellular organization	Vesicular routing, endosomal coordination	Proper signal contextualization	Asynchronous or misaligned signaling
Temporal signal integration	Signal persistence, sequencing	Durable immune activation	Transient or exhausted responses
System-level coherence	Spatial–temporal alignment	Functional immune competence	Structural immune failure

**Table 2:** Structural failure modes versus classical molecular resistance paradigms. Comparison of how structural dysfunction produces immune failure in the absence of detectable molecular defects, highlighting the explanatory limitations of purely reductionist models.

Aspect	Molecular Resistance Models	Structural Failure Framework
Primary cause	Target alteration, pathway inhibition	Loss of spatial–temporal organization
Molecular components	Absent or inhibited	Largely preserved
Signal behavior	Linear, pathway-specific	Non-linear, integrative
Detectability by standard assays	Often detectable	Frequently undetected
Functional outcome	Resistance to intervention	Immune dysfunction without resistance
Implication for interpretation	Target-centric	System-level coherence

**Table 3:** Interpretative implications of structural immune frameworks, illustrating how structural perspectives enhance translational and regulatory legibility for non-pharmacodynamic platforms without introducing new experimental endpoints or requiring target-specific causality.

Evaluation Domain	Conventional Focus	Structural Framework Contribution
Preclinical interpretation	Pathway activation	Organizational coherence
Translational consistency	Target reproducibility	Structural traceability
Immune heterogeneity	Biological noise	Context-dependent architecture
Regulatory assessment	Mechanism-specific	System-level plausibility
Evidence proportionality	Experimental expansion	Interpretative integration

**Table 4:** Comparison of structural determinants with alternative mechanisms of DC dysfunction in cancer.

Mechanism	Key Features	Evidence Strength (Ex Vivo/Clinical)	Representative References	Differentiation from Structural Framework
Genetic alterations	Mutations in *IRF8*, *BATF3*, etc.	Moderate/Strong	[44]	Molecular defect; structural assumes preserved genes
Metabolic reprogramming	Glucose/amino acid depletion, HIF1α	Strong ex vivo/Moderate clinical	[43]	Energy-based; structural focuses on membrane organization
Transcriptional dysregulation	STAT3, Wnt/β-catenin	Strong/Moderate	[55]	Gene expression; structural emphasizes spatial–temporal integration
Epigenetic modifications	Histone/DNA methylation	Moderate/Emerging	[58]	Heritable silencing; structural is biophysical/dynamic
Structural disorganization	Microdomain fragmentation, synapse instability	Strong ex vivo [11,15,18,19,20]/Preliminary clinical correlation [21,27]	[11,15,18,19,20]	Emergent property; explains failure without molecular loss

## Data Availability

No new data were created or analyzed in this study. Data sharing is not applicable to this article.

## Abbreviations

The following abbreviations are used in this manuscript:

DC	Dendritic cell
DEX	Dendritic cell-derived exosome
PLPC	Phospholipoproteic complex
TME	Tumor microenvironment
MHC	Major histocompatibility complex
MHC-I	Major histocompatibility complex class I
MHC-II	Major histocompatibility complex class II
CD	Cluster of differentiation
CD80	Cluster of differentiation 80
CD86	Cluster of differentiation 86
HLA-DR	Human leukocyte antigen DR
IFN-γ	Interferon gamma
IL	Interleukin
IL-10	Interleukin 10
IL-6	Interleukin 6
NAM	New Approach Methodologies
Lo	Liquid-ordered phase
Ld	Liquid-disordered phase
STIP	Structural Traceability Immunophenotyping Platform
PD-1	Programmed cell death protein 1
PD-L1	Programmed death-ligand 1
CCR7	C–C chemokine receptor type 7
ROS	Reactive oxygen species
TCR	T cell receptor
CAR-T	Chimeric antigen receptor T cells
RECIST	Response Evaluation Criteria in Solid Tumors
iRECIST	Immune Response Evaluation Criteria in Solid Tumors
PK	Pharmacokinetics
PD	Pharmacodynamics
